# Ovarian endometrioid carcinoma and clear cell carcinoma: A 21-year retrospective study

**DOI:** 10.1186/s13048-021-00804-1

**Published:** 2021-05-04

**Authors:** Ling Zhou, Liqing Yao, Lin Dai, Honglan Zhu, Xue Ye, Shang Wang, Hongyan Cheng, Ruiqiong Ma, Huiping Liu, Heng Cui, Xiaohong Chang

**Affiliations:** 1 Department of Obstetrics and Gynecology, Center of Gynecologic Oncology, Peking University People’s Hospital, No. 11, South Avenue, Xi Zhi Men, Xicheng District, Beijing, 100044 China; 2 Center of Gynecologic Oncology, Peking University People’s Hospital, Beijing, China; 3 Department of Obstetrics and Gynecology, Department of Obstetrics and Gynecology, Fuding Hospital, Fuding, Fujian Province China; 4 Department of Pathology, Peking University People’s Hospital, Beijing, China

**Keywords:** Ovarian Endometrioid carcinoma, Ovarian clear cell carcinoma, Endometriosis, Prognosis

## Abstract

**Objective:**

This study aimed to identify the clinical characteristics of Chinese patients with ovarian endometrioid carcinoma (EC) and clear cell carcinoma (CCC) and to assess the impact of concurrent endometriosis on this group.

**Methods:**

The present study reviewed the medical records of patients who received initial treatment and a postoperative pathological diagnosis of EC or CCC at our center in China between 1998 and 2018.

**Results:**

Of 211 patients, 73 had pure EC, and 91 had pure CCC, and the remaining 47 had mixed cancer. The proportion of EC and CCC remained stable over past 21 years. The proportion of EC declined with aging and the age of EC onset to incline to the young. And the age of CCC onset had two peaks, namely, 36 and 77 years. After review by the pathologist, the number of endometriosis cases found in the pathological section of the analysis increased to 114, accounting for 54% of patients. As the stage progressed, the appearance of endometriosis became increasingly scarce in pathological sections(*p* = 0.001).

Compared with CCC, EC had a higher frequency of concurrent endometrial cancer (independent endometrial lesions) and estrogen and progesterone receptor expression(*p* = 0.000). And more patients were in premenopausal state in EC group(*p* = 0.040).

In the pure group, multivariate analysis showed that correlation existed between relevance to endometriosis and worse outcomes(*p* = 0.041). In patients with mixed cancer, mixed endometrioid histology was associated with better survival than other subtypes, even with stage III or poorly differentiated tumors(*p* = 0.001).

**Conclusions:**

CCC and EC which are common in ovarian cancer patients who have associated with endometriosis have distinct clinicopathological characteristics. Attention should be paid to ovarian cancer patients with a history of endometriosis and those with concurrent endometriosis in pathological sections.

**Supplementary Information:**

The online version contains supplementary material available at 10.1186/s13048-021-00804-1.

## Introduction

Ovarian cancer is the second most common gynecologic malignancy, with over 90% of cancers arising from epithelial cell s[1, 2]. The most common histology of epithelial ovarian carcinoma (EOC) is papillary serous carcinoma, accounting for 70% of all EOCs in North America, followed by the endometrioid and clear cell histological types, which account for 20–25% and 5–10% of EOCs, respectively, of EOC s[2–5].

Endometriosis is a benign gynecologic disease that is characterized by endometrial glands and stroma occurring outside the uterus. Endometriosis is a risk factor for epithelial ovarian cancer [6, 7], and the overall rate of malignant transformation in endometriosis has been estimated to be 0.3–0.8%, with a relative risk ranging from 1.3 to 1. 9[8]. The relationship between endometriosis and ovarian cancer has been classified as either a transition from endometriotic lesions to invasive ovarian carcinoma or the coexistence of ovarian cancer with endometriosis without a transition ([9]; Roberta B [10].). Sampson defined endometriosis-associated ovarian cancer (EAOC) as endometriosis that was found in the surgical specimen but not in direct continuity with the tumor [11–13]. It is also well known that tumors associated with endometriosis are confined to specific subcategories of disease, namely, endometrioid carcinoma (EC) and clear cell carcinoma (CCC) ([14]; R. B [15].).

The prognosis of early ovarian CCC is good, but late-stage CCC is known to be less sensitive to platinum-based front-line chemotherapy and to be associated with a worse prognosis than serous adenocarcinoma or endometrioid adenocarcinoma during the same period [16–19]. It is generally believed that the prognosis of ovarian EC is good, but there are still some patients with a poor prognosis (drug resistance, recurrence or even death), which has also been reported in the literature [20]. There are still many unresolved problems with CCC and EC as tumors closely related to endometriosis. To obtain further insight into these endometriosis-related cancers, we reviewed and analyzed the clinical data of patients with CCC and EC in a single center over the past 20 years. We aimed to summarize the clinical experience and provide guidance for clinical work.

## Materials and methods

### Patients and clinical information

With ethical approval (No. 2020PHB212–01) given for ovarian cancer specimen collection with annotated clinical information, all patients with primary EOC who were treated at Peking University People’s Hospital from 1998 to 2018 were reviewed. Individual informed patient consent was not sought, as these data were routinely obtained and used as part of these patients’ clinical care. The women were followed until December 2019 or until they died of ovarian cancer or other conditions, whichever happened first.

**Fig. 1:**
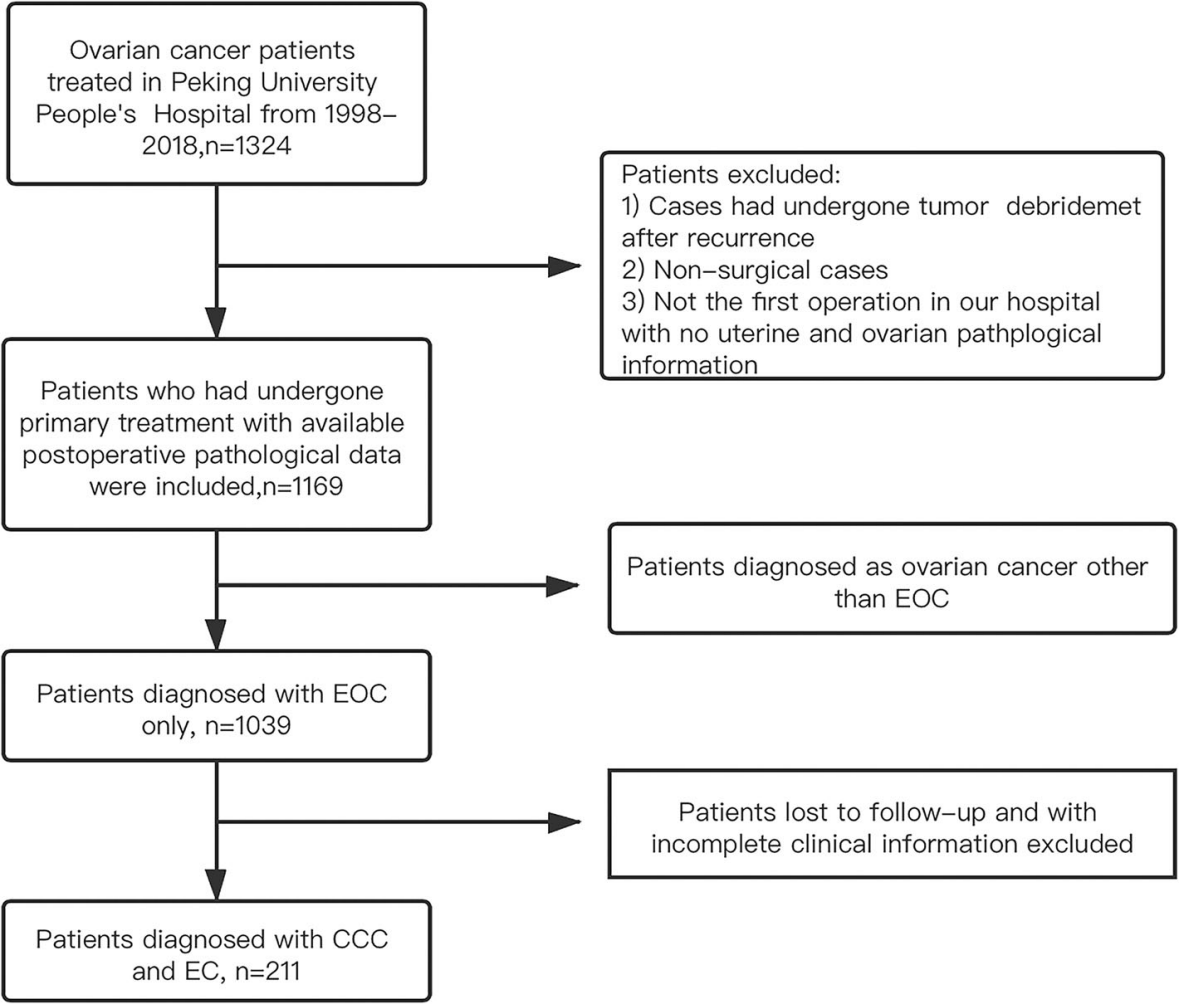
Flowchart of included patients. CCC, clear cell carcinoma; EC, endometrioid carcinoma; EOC, epithelial ovarian cancer

Data from electronic medical records were retrospectively reviewed. Two pathologists reviewed the pathology details to determine whether there was concurrent endometriosis. We screened patients according to the established criteria (see the detailed process in Fig. 1), and patients with complete clinical data and follow-up information were included. The pathological staging was checked, and patient was restaged based on the International Federation of Obstetrics and Gynecology (FIGO) staging standard (according to the 2014 FIGO staging standard to restage ovarian cancer and according to the 2009 FIGO staging standard to restage concurrent endometrial cancer).

The following information was collected: age at diagnosis, histology, history of past illnesses (hypertension/diabetes/endometriosis/breast cancer/immune system disease), FIGO stage, fertility history, first symptoms, blood cancer antigen-125 (CA-125) level (before and after surgery), tumor size and side, surgical procedure, tumor grade, surgical debulking status, endometrial pathology (concurrent endometrial lesions), chemotherapy regimen, objective response to chemotherapy, progression-free survival and overall survival.

### Criteria for defining factors

Endometrial lesions included endometrial cancer and endometrial dysplasia. If there was endometrial cancer by uterine pathology, the Scully criteria were used to distinguish between dual primary and metastatic disease (the full description of the criteria is provided in Supplementary Table) [21]. Endometriosis-related patients were defined as patients showing endometriosis by pathology or having a history of endometriosis. Progression-free survival (PFS) was defined as the time interval from the date of primary surgery to the date of disease progression and/or recurrence. Overall survival (OS) was defined in months as the date of the primary surgery to the date of death or censoring at the date of last contact. To define residual disease status after primary debulking surgery, the largest diameter of residual disease was measured and categorized as follows: no residual disease (R0), 0.1–1 cm residual disease (R1), and > 1 cm residual disease.

### Statistical analysis

Summary statistics were used to describe the data. Medians (ranges) or means (standard deviations) were used for continuous variables. After a normal distribution was confirmed with the Shapiro-Wilks test, the Mann-Whitney U test was used to compare median values, and Student’s t-test was used to compare mean values. Categorical variables are presented as frequencies (percentages). Fisher’s exact test or the χ2 test were used to analyze the distributions of characteristics according to their associations with endometriosis. Survival curve analyses were performed with the Kaplan-Meier method, and comparisons were performed using the log-rank test. A Cox proportional hazards model was used to perform univariate and multivariate analyses to evaluate the prognostic significance of the association with endometriosis and other clinicopathological features. Multivariate *p*-values were used to present the significance of each feature. To quantify the correlation between survival time and each independent feature, a 95% confidence interval (CI) was used. All *p*-values were 2-sided, and p-values less than 0.05 were considered statistically significant.

The Joinpoint Regression Program 4.6.0.0 provided by the National Cancer Institute was used to determine potential changes in the temporal trends in the incidence rate. The trends in the histological subtypes of ovarian cancer were examined for every calendar year or every patient age. Linear segmented regression analysis was utilized for the model. Statistical analyses were performed using SPSS 26.0 (IBM), GraphPad Prism 8.0 and R 3.0.6 (R Foundation, Vienna, Austria; http://www.R-project.org).

## Results

### Patient demographics

From 1998 to 2018, there were 1324 patients with ovarian cancer who were admitted to Peking University People’s Hospital. According to the exclusion criteria, 211 patients who were diagnosed as having clear cell or endometrioid histology were investigated (the patient selection schema is shown in Fig. 1). Of 211 patients, 73 had pure EC, and 91 had pure CCC. There were 47 cases of mixed cancers of other histological types, such as the serous and mucinous types.

**Fig. 2:**
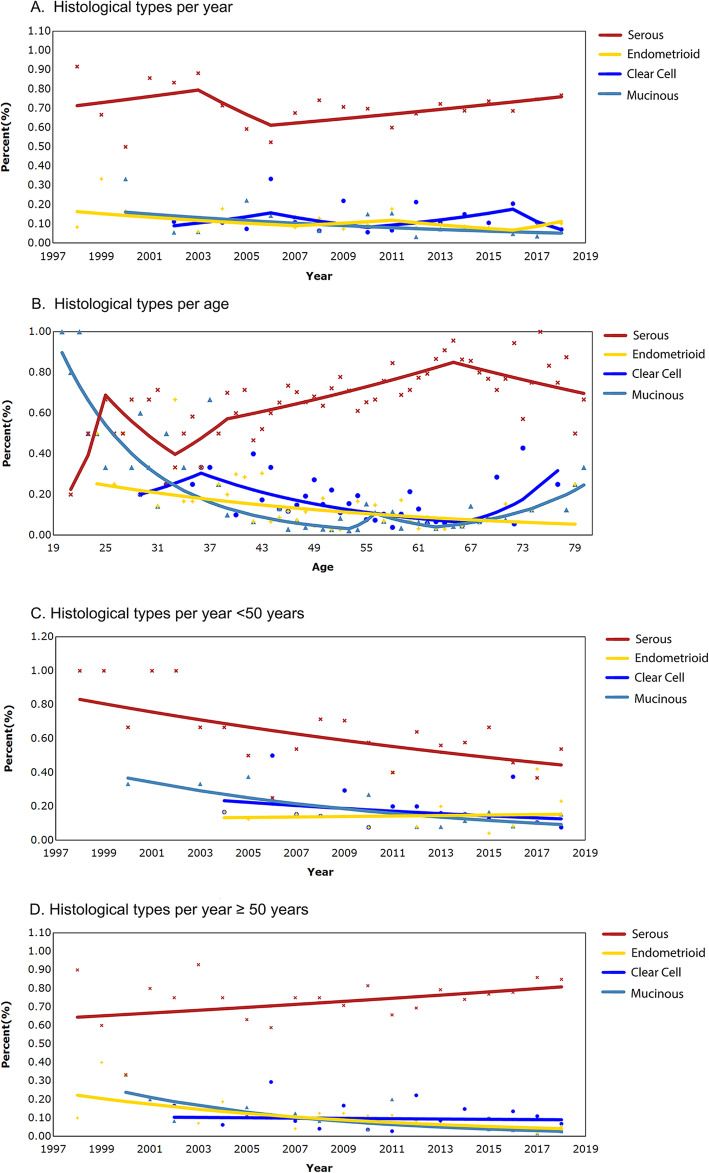
Time and temporal trend of ovarian cancer histology with age. Lines are estimated values for modeling, and points represent actual data. **a** The annual percentage of each histological subtype among the four major primary epithelial ovarian cancers is shown. **b**-**d** At diagnosis, the four histological subtypes of epithelial ovarian cancer were stratified by age. **c** < 50 years old; (D) ≥ 50 years old

Among pure EC patients, the most common histological subtype was serous (669/938, 71.3%), followed by clear cell (104/938, 11.1%), endometrioid (89/938, 9.5%), and mucinous (76/938, 8.1%). Figure 2 show the time-specific trends and age-specific trends for each histological type. From 1998 to 2018, the proportions of ovarian cancer subtypes remained stable (Fig. 2a). Overall, the proportion of serous ovarian cancer gradually increased with increasing age while the proportion of EC declined with aging (Fig. 2b). The age of onset of CCC had two peaks, namely, 36 years and 77 years (Fig. 2b).

One can interpret the data as indicating that serous ovarian cancer patients were getting older, while patients with endometrioid ovarian cancer were getting younger over time (Fig. 2 C-D). Among women aged < 50 years in the cohort, the incidence rate of EC increased between 1998 and 2018 (rising by nearly 1.02% per year; Fig. 2c). The incidence rates of the three cancer types decreased between 1998 and 2018 (clear cell, declined by 4.28% per year; serous, declined by 3.08% per year; mucinous, declined by 7.32% per year. Figure 2c). For people ≥50 years old, the frequency of serous cancer increased slightly in 1998 and 2018, while that of the other three types showed a downward trend (serous, rising by nearly 1.14% per year; clear cell, declining by 0.86% per year; endometrioid, declining by 8.01% per year; mucinous, declined by 11.28% per year. Figure 2d).

### Total clinical and morphological features

The detailed data are presented in Tables 1-2. The mean age at diagnosis of the entire population was 51.9 ± 10.9 years (range, 24–79 years), and 47.9% (101/211) of the women were postmenopausal.

**Table 1:** Total clinical characteristics

Clinicopathological Factor	Number(%)/Median(P_25,_P_75_)
EC	73 (34.6%)
CCC	91 (43.1%)
Mix	47 (22.3%)
Mixed with EC	31 (14.7%)
Mixed with CCC	13 (6.2%)
Both	3 (1.4%)
EMs in Pathological Section	114 (54.0%)
Past History of EMs	16 (7.6%)
Endometrial cancer	26 (12.3%)
Concurrent with pure EC	23 (10.9%)
Concurrent with pure CCC	1 (0.47)
Parity	
> 1	170 (80.6%)
0	41 (19.4%)
Past History of Hypertension	46 (21.8%)
Past History of Diabetes	18 (8.5%)
Overweight (BMI > 24)	89 (42.4%)
Dysmenorrhea	49 (23.2%)
Post-menopause	101 (47.9%)
Breast cancer	8 (3.8%)
Immune System Disease	7 (3.3%)
Initial Signs/Symptoms	
Abdominal Pain	42 (19.9%)
Abdominal Distension	40 (19.0%)
Abdominal Mass	17 (8.1%)
Asymptomatic	68 (32.2%)
Vaginal Bleeding	15 (7.1%)
Painful Menstruation	2 (0.92%)
Others^a^	27 (12.8%)
CA-125 level^b^	
Pre-operation	96.0 (31.4, 378.0)
Post-operation	42.5 (19.1, 99.34)

^a^Others symptoms contain weariness,emaciation, lower extremity edema, abnormal vaginal discharge, irregular menstruation and change of menstrual volume

^b^Because it was not normally distributed, statistics are indicated with medians and quartiles

**Table 2:** Total characteristics of therapy and prognosis

Clinicopathological Factor	Number
Operation	211 (100%)
Complete Surgical Staging	114 (54.0%)
Maximal Resection	97 (46.0%)
Residual Disease	
R0	142 (67.3%)
R1(< 1 cm)	13 (6.2%)
1–2 cm	7 (3.32%)
> 2 cm	8 (3.79%)
Record not found	41 (19.4%)
Second Operation	11 (5.25%)
Abdominal Dropsy	
0	78 (37.0%)
< 500 ml	81 (38.4%)
> 500 ml	34 (16.1%)
Record not found	18 (8.5%)
Lymph Nodes Excision (LMN)	188 (89.1%)
No	23 (10.9%)
Pelvic Region Only	2 (0.9%)
Pelvic & Abdominal region	186 (88.2%)
Surgery to Conserve Fertility	8 (3.8%)
Chemotherapy Regimens	177 (83.9%)
Platinum-based chemotherapy (PBC)	174 (82.3%)
Platinum-free chemotherapy	3 (1.4%)
TC	150 (71.1%)
Others	27 (13.0%)
FIGO stage	
I	114 (54.0%)
II	39 (18.5%)
III	54 (25.6%)
IV	4 (1.9%)
Chemotherapy Treatment Course	
< 6	39 (18.5%)
≥ 6	138 (65.4%)
Death	29 (13.7%)
Relapse	48 (22.7%)

The common symptoms at initial presentation were sequentially palpable mass, abdominal pain, incidental finding, abdominal distension and irregular vaginal bleeding. A normal preoperative serum CA-125 value was observed in 27.5% (58/211) of patients. The distribution of FIGO stage was as follows: Stage I, 54% (114/211); Stage II, 18.5% (39/211); Stage III, 25.6% (54/211); and Stage IV, 1.9% (4/211). There were 16 patients with a previous history of endometriosis. In the initial records, endometriosis was found by pathology in 41 patients. After review by the pathologist, the number of endometriosis cases found in the pathological section of the analysis increased to 114, accounting for 54% of patients. From the early stage to the late stage, the frequency of endometriosis by pathology gradually decreased (via the linear by linear association method, *p* = 0.001).

In this series, 114 (54.0%) patients received comprehensive staging surgery, and 97 (46.0%) patients received cytoreductive surgery. A total of 142 (67.3%) patients had no residual lesions, and 13 (6.2%) patients had residual lesions less than 1 cm in size. In total, 177 (83.9%) patients received chemotherapy, among which 174 (82.3%) received PBC.

### Pure clear cell and endometrioid carcinoma

The present study found that the age at diagnosis was 50.0 ± 11.7 (range 24 to 79) years in the EC group and 52.6 ± 9.5 years (range 32 to 77) in the CCC group (see details in Table 3). Compared with CCC, EC has a higher frequency of concurrent endometrial cancer and a higher frequency of independent ovarian and endometrial lesions (including endometrial cancer and precancerous lesions, *p* = 0.000). According to the standard of Scully et al. [22], patients with synchronous primary cancer of the endometrium and ovary accounted for 20.5% (15/73) of the EC group, while they accounted for only 1.1% of the CCC group (*p* = 0.000). The proportion of premenopausal women was higher in the EC group (*p* = 0.040). EC were predominantly positive for estrogen receptor (ER) and progesterone receptor (PR), but CCC exhibited lower ER and PR expression (*p* = 0.000) (Table 4).

**Table 3:** Mean age of each group

Group	Age at diagnose (range)
All Patients	51.8 ± 10.9 (24–79)
Pure Cancer	51.4 ± 10.6 (24–79)
EC	50.0 ± 11.7 (24–79)
CCC	52.6 ± 9.5 (32–77)
Mixed Cancer	53.4 ± 12.0 (28–75)

*CCC* Clear cell carcinoma; *EC* Endometrioid carcinoma

**Table 4:** The results of immunohistogical staining of ER and PR of pure cancer

	Number
ER	62
EC	51 (51/62, 82.3%)
CCC	11 (11/62, 17.7%)
PR	55
EC	49 (49/55, 89.1%)
CCC	6 (6/55, 10.9%)

*ER* Estrogen receptor; *PR* Progesterone receptor; *CCC* Clear cell carcinoma; *EC* Endometrioid carcinoma

**Fig. 3:**
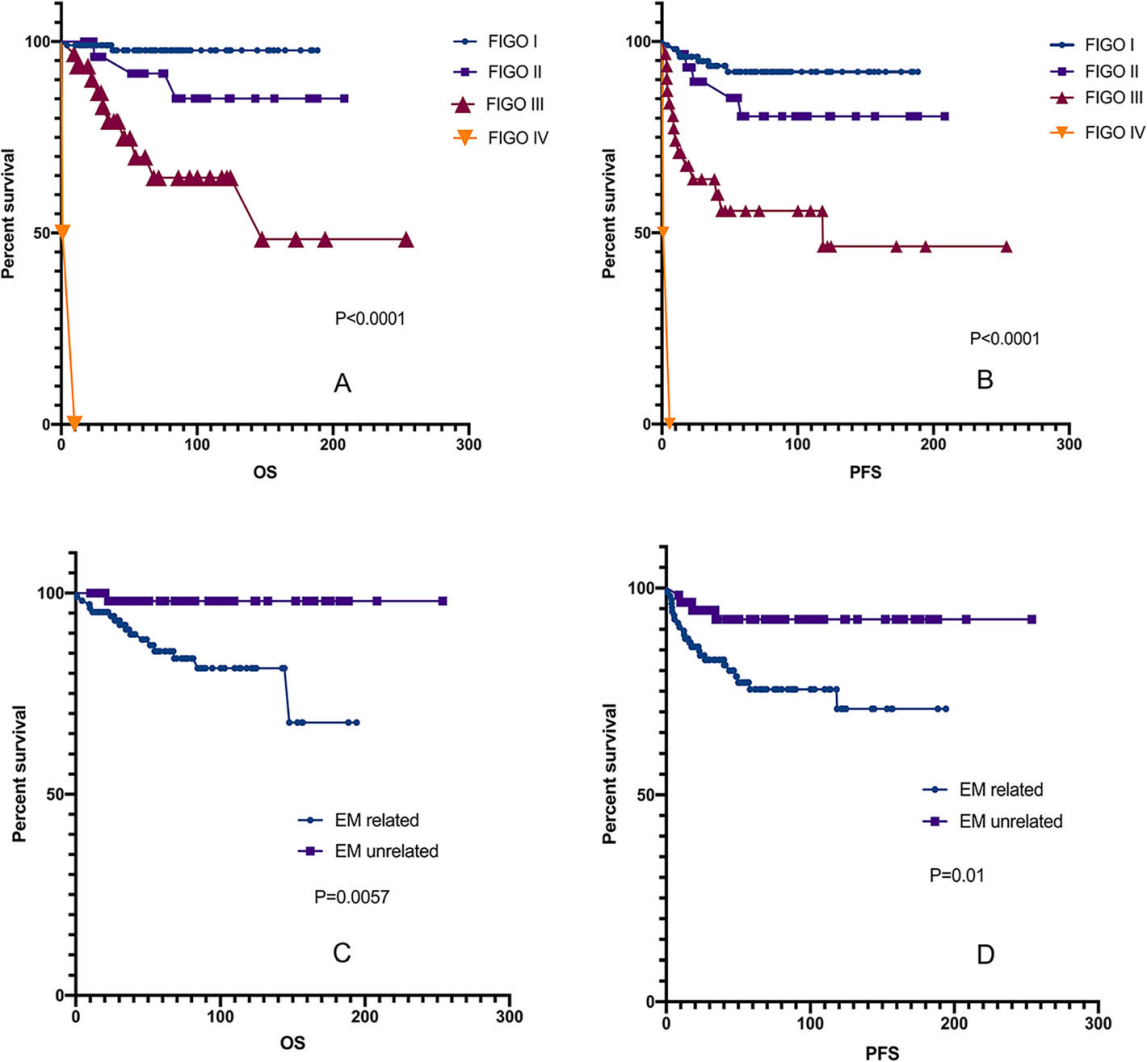
Kaplan-Meier curve for OS and PFS in patients with pure cancer. No statistically significant difference in EC and CCC group. Kaplan-Meier survival curves showing the effects of FIGO stage (**a** and **b**) and relevance to EM (**c** and **d**). PFS, progression-free survival PFS, progression-free survival; OS, overall survival. CCC, clear cell carcinoma; EC, endometrioid carcinoma; EM, endometriosis

During the follow-up period, 17 patients (10.4%) died, and 28 patients (17.1%) experienced relapses. The 5-year OS and PFS rates of CCC patients were 88.0 and 89.9%, respectively. The 5-year OS and PFS rates of EC patients were 92.1 and 78.4%, respectively. The difference in prognosis between EC and CCC was not significant (*p* = 0.333. Figure 3 a-b).

Survival analysis of 164 pure EOC patients with EC or CCC was performed by univariate analysis using the Kaplan-Meier method. The results showed that the factors that influenced the prognosis of pure EOC patients with EC or CCC included FIGO stage, lymphadenectomy, residual lesions, parity history, association with endometriosis and CA-125 level before and after surgery (*p* < 0.05). Survival analysis revealed significant differences in PFS and OS between patients whose disease was related to endometriosis compared to those whose disease was unrelated to endometriosis. The Kaplan-Meier curve for PFS showed a survival advantage for patients whose disease was unrelated to endometriosis (*p* = 0.01; Fig. 3.f). The results for OS indicated that patients whose disease was unrelated to endometriosis had better survival outcomes than patients whose disease was related to endometriosis (*p* = 0.0057; Fig. 3.e). Rambau P et al.(P [23].) reported that the expression of ER and PR was significantly associated with longer ovarian cancer-specific survival, but no association was found in this study.

**Fig. 4:**
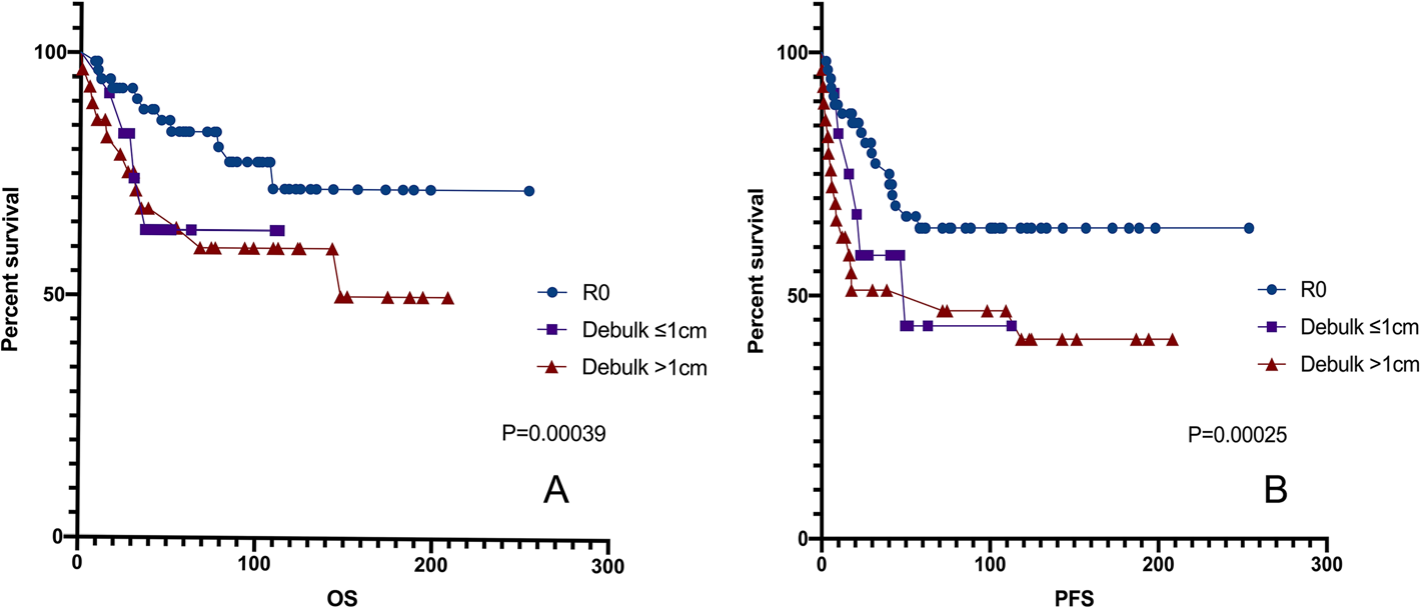
Kaplan-Meier curve for (**a**) OS and (**b**) PFS in stage II-IV patients with pure cancer. PFS, progression-free survival; OS, overall survival. R0, no residual disease

In the multivariate analysis of clinicopathologic variables among the entire cohort of patients, disease related to endometriosis remained a significant prognostic factor for PFS (*p* = 0.001; Table 5). FIGO stage, which was previously shown to be a prognostic factor in EOC, remained a significant prognostic factor for PFS and OS in this study cohort (Fig. 3.c-d). Additionally, the postoperative CA-125 level was significant in the multivariate analysis for PFS. Both the univariate and multivariate analyses showed that in advanced ovarian cancer (stage II-IV), residual lesions are an independent prognostic factor (Fig.4; Table 5).

**Table 5:** Multivariate Cox proportional hazards analysis for PFS and OS used to adjust risk associated prognostic clinical features

Pure group (*n* = 164)	PFS		OS	
	HR (95%CI)	*p*-value	HR (95%CI)	*p*-value
Parity	0.906 (0.508–1.615)	0.738	/	/
CA-125(before)	1.000 (0.999–1.000)	0.595	1.646 (0.403–6.725)	0.487
CA-125(after)	1.002 (1.000–1.005)	0.044	/	/
FIGO	1.722 (0.990–2.994)	0.054	3.981 (1.821–8.706)	0.001
EM-related	4.014 (1.056–15.262)	0.041	0.925 (0.272–3.146)	0.047
Residual disease	0.915 (0.475–1.766)	0.792	1.389 (0.823–2.346)	0.219
Lymph Nodes excision	0.325 (0.078–1.346)	0.325	0.925 (0.272–3.146)	0.900
Mixed group (*n* = 47)	PFS		OS	
	HR (95%CI)	*p*-value	HR (95%CI)	*p*-value
CA-125(before)	1.000 (0.999–1.001)	0.835	1.000 (1.000–1.001)	0.185
CA-125(after)	1.003 (0.998–1.008)	0.235	/	/
FIGO	1.934 (0.211–17.700)	0.023	1.880 (0.699–5.059)	0.211
EM-related	/	/	/	/
Residual disease	1.490 (0.544–4.087)	0.544	1.030 (0.508–2.086)	0.935
Mixed with EC	0.070 (0.014–0.347)	0.001	0.425 (0.107–1.684)	0.223
Appendix excision	4.460 (0.350–56.794)	0.249	0.228 (0.041–1.258)	0.090
Lymph Nodes excision	1.934 (0.211–17.700)	0.559	1.030 (0.508–2.086)	0.935
2–4 stage of patients with pure cancer		PFS		
		HR (95%CI)	*p*-value	
FIGO		2.918 (1.022–8.326)	0.045	
R0		0.287 (0.110–0.749)	0.011	

*CA* Cancer antigen; *CA-125(before)* CA-125 level before surgery; *CI* Confidence interval; *FIGO* International Federation of Gynecology and Obstetrics; *HR* Hazard ratio; *OS* Overall survival; *PFS* Progression-free survival. R0, Achieving optimal cytoreduction and/or removal of all macroscopic disease

### Mixed carcinoma

There were 47 cases of mixed cancers of other histological types, including 31 cases of cancers mixed with ECs, 13 cases of cancers mixed with CCCs, and three cases of cancers mixed with ECs and CCCs (see details in Tables 1-2). The mucinous type always appeared with the endometrioid histological type rather than with CCC (*p* = 0.000).

**Fig. 5:**
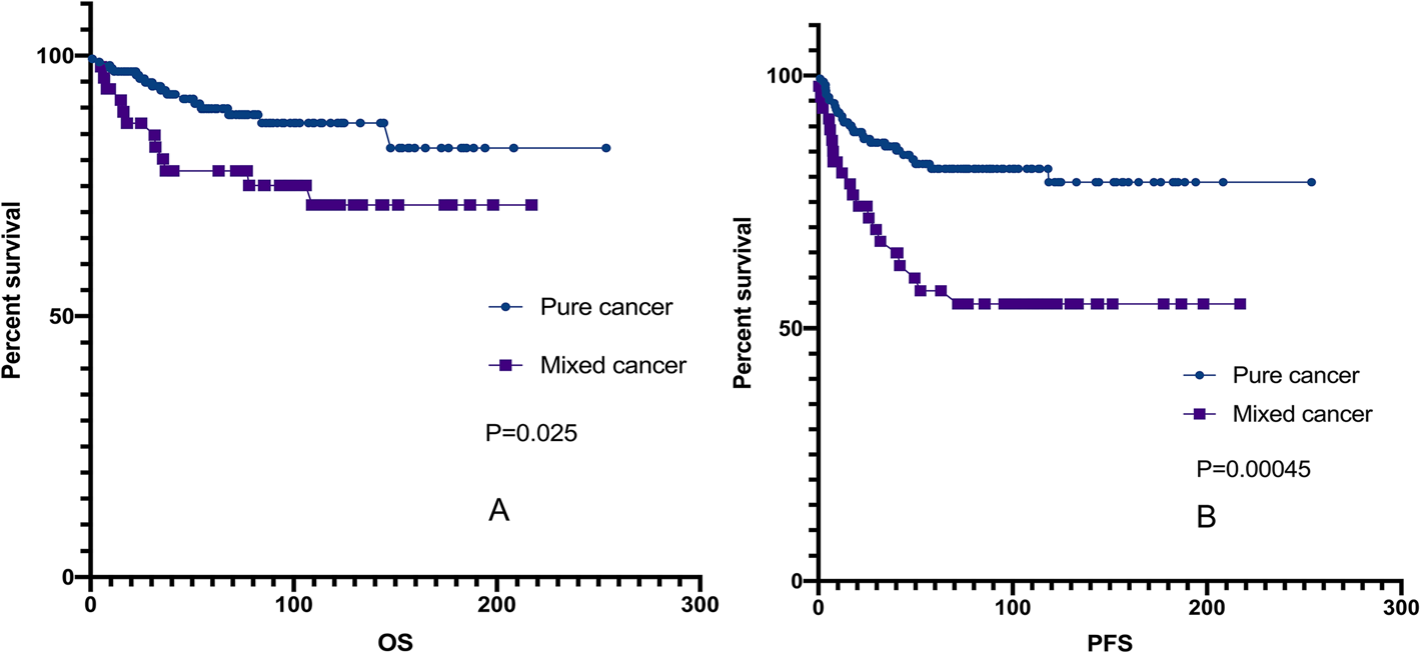
Kaplan-Meier curve for (**a**) OS and (**b**) PFS in all patients. PFS, progression-free survival; OS, overall survival

**Fig. 6:**
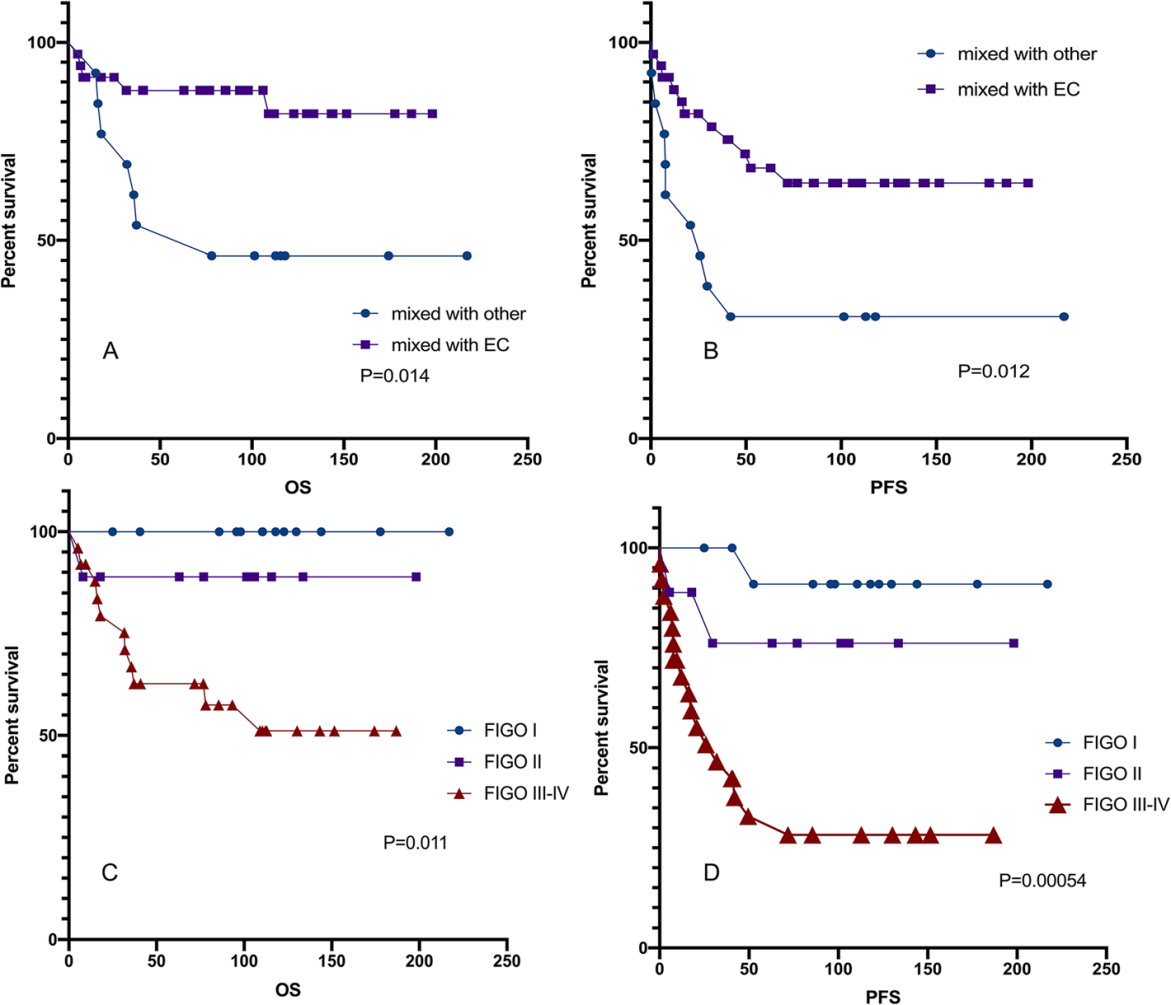
A comparison of PFS and OS in mixed cancer patients with different variables. Kaplan-Meier survival curves showing the effects of FIGO stage (**c** and **d**) and mixed EC (**a** and **b**). PFS, progression-free survival; OS, overall survival. CCC, clear cell carcinoma; EC, endometrioid carcinoma

During the follow-up period, 12 patients (42.6%) died, and 20 patients (25.5%) experienced relapses. The 5-year OS and PFS rates of mixed carcinoma were 77.9 and 57.4%, respectively. The difference in prognosis between mixed carcinoma and pure carcinoma was significant (*p* < 0.05, Fig.5). The results for OS and PFS indicated that patients with pure cancer had significantly better survival outcomes (Fig.5). Univariate and multivariate analyses showed that FIGO stage and cancer mixed with endometrioid histology had a statistically significant impact on the prognosis of patients with mixed cancer (Fig.6; Table 5). Mixed endometrioid histology was associated with better survival than serous adenocarcinoma of the ovary, even with stage III or poorly differentiated tumors (p = 0.000).

## Discussion

### Ovarian clear cell carcinoma and endometrioid carcinoma

This study systematically described CCC and EC data from a single center in China obtained from 1998 to 2018. The distribution and age structure of the four major histological subtypes of pure ovarian cancer in China are more similar to those of the United States than to those of Japan, another Asian country [24]. There was a tendency to young people for patients with EC in age. It might be because continuous development of human society, people’s bearing attitudes are constantly changing, and urbanization has transformed people’s ideas on family planning. Women have fewer pregnancy and childbirths, and there are more alternative contraceptive methods, such as the use of short-acting combined contraceptives. To further determine the distribution and specific trends of ovarian cancer patients in China, multicenter, large-sample research is needed in the future.

Survival analysis showed that FIGO stage is an important prognostic factor in pure cancer or mixed cancer. The residual lesion size, which is generally considered to be an independent prognostic factor, is still important in advanced pure ovarian cancer (stage II-IV). The pure cancer group had a better overall prognosis than the mixed group, and among mixed cancers, EC was always associated with a better prognosis. In another single-center study, Dawn J. Story et al. found that despite their similar response rates to platinum-based chemotherapy (PBC), endometrioid ovarian cancer has a better survival outcome than ovarian serous adenocarcinoma [25].

Two histologic types, CCC and EC, are common histological types of ovarian cancer in patients who have associated endometriosis. However, both tumor types have distinct clinicopathological characteristics and molecular phenotypes. The high incidence of combined endometrial disease in the EC group distinguishes EC from CCC. Studies have shown that EC subtypes have a much higher chance of concurrent endometrial cancer than other histological subtypes of epithelial ovarian cancer in the same period [21]. Our data also indicated that 12.3% (9/73) of patients with EC had coexisting endometrial atypical hyperplasia and that 20.5% (15/73) of patients with EC had synchronous endometrial cancer, which should be seriously taken into account, particularly for patients with the desire to undergo fertility-sparing surgery. A comprehensive evaluation of the endometrium might be necessary.

In terms of morphological characteristics, ECs show broad morphological similarities to their endometrial counterparts. Overall, the most common molecular alterations in EC include mutations in CTNNB1 (31–53.3%), PIK3CA (15–40%), ARID1A (30%), and PPP2R1A (7–16.6%) ([26–31]; P. F [32].). Largely, these events are similar to those that have been reported for the more common endometrial EC. Similar to their endometrial counterparts, EC seems to be classifiable into molecular subgroups that correlate with survival [33]. Add all of these together and it is not hard to see that EC is closer to endometrial cancer in all aspects. Recent studies based on targeting and exon sequencing have confirmed that there is a clonal correlation between ovarian and endometrial primary cancers. The two are not absolutely unrelated; it may be the spread from one site to another. The author believes that EC of the ovary may be an ovarian implant of indolent endometrial carcinoma and puts forward the following hypotheses: 1) The metastasis may be caused by retrograde progression of the diseased endometrium through the fallopian tube rather than blood or lymph node metastasis; 2) There is the possibility of “pseudometastasis”, namely, cells detach from the primary lesion without undergoing apoptosis, spread through open spaces, and only recolonize the unique microenvironment without the ability to extensively metastasize [34, 35]. It seems that EC is another form of endometrial cancer that grows in the ovaries. Therefore, when studying the pathogenesis of EC and CCC, there are two possibilities to consider as the determining factor—bad endometriosis or a bad endometrium. Perhaps treatment of EC that is analogous to endometrial cancer will become a new breakthrough.

EC is predominantly positive for ER, but CCC exhibits lower ER expression. It has been [36] proposed a model postulating that additional events, particularly deletion of ER expression, are required for CCC lesion progression. CCC pathogenesis may be a model to study the progression from estrogen-dependent to estrogen-independent disease, allowing the design of new strategies targeting the hormone response, thereby modifying disease outcome. Therefore, loss of estrogen function may be a turning point in CCC development. There are still many problems with CCC and EC in current clinical practice, but the treatment options available are likely similar to serous histological subtypes. The 2016 National Comprehensive Cancer Network (NCCN) guidelines listed hormone therapy as a postoperative adjuvant treatment option for low-grade EC. This shows that the application of treatments that target ER and PR in the treatment of EC and CCC is gradually being valued. However, further research is needed to prove the role of ER and PR in the development of endometriosis-related ovarian cancer.

Recently, sophisticated proteomic tracing studies have suggested that ovarian endometrioid adenocarcinomas arise from secretory cells of endometriosis or the endometrium, while ovarian clear cell adenocarcinomas arise from ciliated cells. Importantly, it is hypothesized that the unique cellular environment dictates the development of ciliated or secretory cells, which then gain mutations to become malignant [37]. Indeed, it is worth exploring the following: Why are there two different types from the same origin (if both CCC and EC originate from endometriosis)?

### The relationship between endometriosis and CCC/EC

After review by the pathologist, the number of endometriosis cases found in the pathological section rose from 19.4 to 54.0% of total cases (from 41 to 114 patients). This suggests that the proportion of EC and CCC coexisting with endometriosis was previously underestimated. The reasons for the differences before and after the pathological review may be as follows: 1) tumor cells are so aggressive that they invade and destroy most or all endometriotic tissues; 2) because pathology reports are used for the diagnosis of malignant tumors, the reporter focuses on malignant tumors rather than endometriosis; and 3) we focus more on the tumor site when taking pathological sections. In clinical work, it is very difficult to obtain specimens of endometriosis, tumor tissues and transitional tissues all at once.

In this study, as the stage progressed, the appearance of endometriosis became increasingly scarce in pathological sections (*p* = 0.001). The probability of endometriosis coexisting in early disease is higher, which is consistent with the conclusion drawn by Kim HS et al. [38]. In patients with advanced cancer, the low frequency of endometriosis might be because the endometriotic tissue was “burned out”, that is, completely transformed into cancer tissue. It is also possible that malignant tumor cells proliferated rapidly and invaded endometriotic tissues and other benign tissues. Thus, the absence of endometriosis in the pathological sections of EC and CCC does not mean that it does not exist in the specimen. It may be that the pathologist did not report the endometriosis (it was not taken seriously) or that endometriosis was “hidden” under the tumor tissue.

Several studies have reported that endometriosis has no effect on the prognosis of EAOC [39, 40], and we did find that there was no association between the presence of endometriosis and the prognosis of ovarian CCC or EC (*p* = 0.091). Considering that the absence of endometriosis does not mean that it never existed, we consider a history of endometriosis and cooccurrence of endometriosis as a whole to be a clinical pathological factor. As a result, we found that patients with endometriosis had worse outcomes. This may be explained by the abnormal immune regulation system and aberrant pelvic microenvironment in patients with endometriosis [41]. As a benign disease behaving like a malignant tumor, endometriosis can invade tissues and spread elsewhere. With abnormal secretion of immune regulatory factors and abnormal activation of the complement system [41], immune escape occurs and creates a favorable environment for recurrence. This is a reminder that a history of endometriosis in ovarian cancer patients should be taken seriously and that long-term management and close follow-up are necessary.

To our knowledge, this is the largest retrospectively collected pure and mixed ovarian clear cell and EC set in China. We have discussed the relationship between endometriosis and EC or CCC, aiming to provide some guidance for clinical work. Attention should be paid to ovarian cancer patients with a history of endometriosis and those with concurrent endometriosis in pathological sections. This study also has some limitations. Multicenter, large-sample prospective clinical research is necessary in the future.

## Supplementary Information

**Additional file 1.**

## Data Availability

The dataset supporting the conclusions of this article is included within the article and its additional files.
