# Supplementary material for: Ovarian endometrioid carcinoma and clear cell carcinoma: A 21-year retrospective study
Source: J Ovarian Res. 2021 May 4;14:63. doi: 10.1186/s13048-021-00804-1 (PMC8094516; doi:10.1186/s13048-021-00804-1)
Supplement: Supplementary file 1 — Additional file 1. [file 13048_2021_804_MOESM1_ESM.docx]

Supplement Table.1.

| Independent primary tumors |
| --- |
| 1. Histologic dissimilarity of the tumors |
| 2. No or only superficial myometrial invasion of endometrial tumor |
| 3. No vascular space invasion of endometrial tumor |
| 4. Atypical endometrial hyperplasia additionally present |
| 5. Absence of other evidence of spread of endometrial tumor |
| 6. Ovarian tumor unilateral (80%–90% of cases) |
| 7. Ovarian tumor located in parenchyma |
| 8. No vascular space invasion, surface implants, or predominant hilar location in ovary |
| 9. Absence of other evidence of spread of ovarian tumor |
| 10. Ovarian endometriosis present |
| 11. Different ploidy of DNA indices, if aneuploid, of the tumors |
| 12. Dissimilar molecular genetic or karyotypic abnormalities in the tumors |

From Scully et al. Tumors of the Ovary, Maldeveloped Gonads, Fallopian Tube, and Broad Ligament, Atlas of Tumor Pathology, Armed Forces Institute of Pathology/American Registry of Pathology, Washington D.C., 1998.
